# The Complexity of TET2 Functions in Pluripotency and Development

**DOI:** 10.3389/fcell.2020.630754

**Published:** 2021-01-18

**Authors:** Vera Garcia-Outeiral, Cristina de la Parte, Miguel Fidalgo, Diana Guallar

**Affiliations:** ^1^Stem Cells and Human Diseases Group, Department of Physiology, Center for Research in Molecular Medicine and Chronic Diseases, Universidade de Santiago de Compostela, Santiago de Compostela, Spain; ^2^Epitranscriptomics and Ageing Group, Department of Biochemistry and Molecular Biology, Center for Research in Molecular Medicine and Chronic Diseases, Universidade de Santiago de Compostela, Santiago de Compostela, Spain

**Keywords:** TET2, epigenetic, epitranscriptomic, pluripotency, reprogramming, development, 5hmC

## Abstract

Ten-eleven translocation-2 (TET2) is a crucial driver of cell fate outcomes in a myriad of biological processes, including embryonic development and tissue homeostasis. TET2 catalyzes the demethylation of 5-methylcytosine on DNA, affecting transcriptional regulation. New exciting research has provided evidence for TET2 catalytic activity in post-transcriptional regulation through RNA hydroxymethylation. Here we review the current understanding of TET2 functions on both DNA and RNA, and the influence of these chemical modifications in normal development and pluripotency contexts, highlighting TET2 versatility in influencing genome regulation and cellular phenotypes.

## Introduction

TET2 belongs to the Ten-eleven-translocation (TET) family of proteins, which also includes TET1 and TET3. The expression of the three TET enzymes differs during early embryonic development, with TET3 mostly restricted to oocytes and zygotes, while TET1 and TET2 are highly expressed later in preimplantation embryos (Gu et al., 2011; Iqbal et al., 2011; Wossidlo et al., 2011). TET proteins have been extensively characterized as α-ketoglutarate and Fe(II)-dependent dioxygenases capable of catalyzing the iterative oxidation of 5-methylcytosine (5mC) to 5-hydroxymethylcytosine (5hmC) (Tahiliani et al., 2009; Ito et al., 2010), 5-formylcytosine (5fC) and 5-carboxylcytosine (5caC) (Ito et al., 2011) on DNA. Importantly, 5hmC is now not only considered a demethylation intermediate but also an epigenetic mark by itself. TET2 plays thus key roles in shaping the methylome of a cell and establishing novel 5hmC-enriched genomic regions, including enhancers, for chromatin and transcriptional regulation. Moreover, recently we and others have shown that TET2 can also oxidize m^5^C (to distinguish it from 5mC in DNA) in RNAs of ESCs and during myelopoiesis for the regulation of RNA stability (Guallar et al., 2018; Shen et al., 2018; He et al., 2020; Lan et al., 2020) Here we review the current understanding of TET2 functions, providing an overview of its roles in development and pluripotency contexts. Although multiple studies have approached 5hmC function through double and triple *Tet1/2/3* depletion (Dawlaty et al., 2013, 2014; Lu et al., 2014; Kang et al., 2015; Li et al., 2016; Verma et al., 2018; Charlton et al., 2020) we focus on *Tet2* single mutants to try to better dissect TET2 specific functions on both DNA and RNA by integrating structure and function, providing a comprehensive overview of this versatile epigenetic and epitranscriptomic regulator.

## TET2 Protein Structure and Function

**Figure 1:**
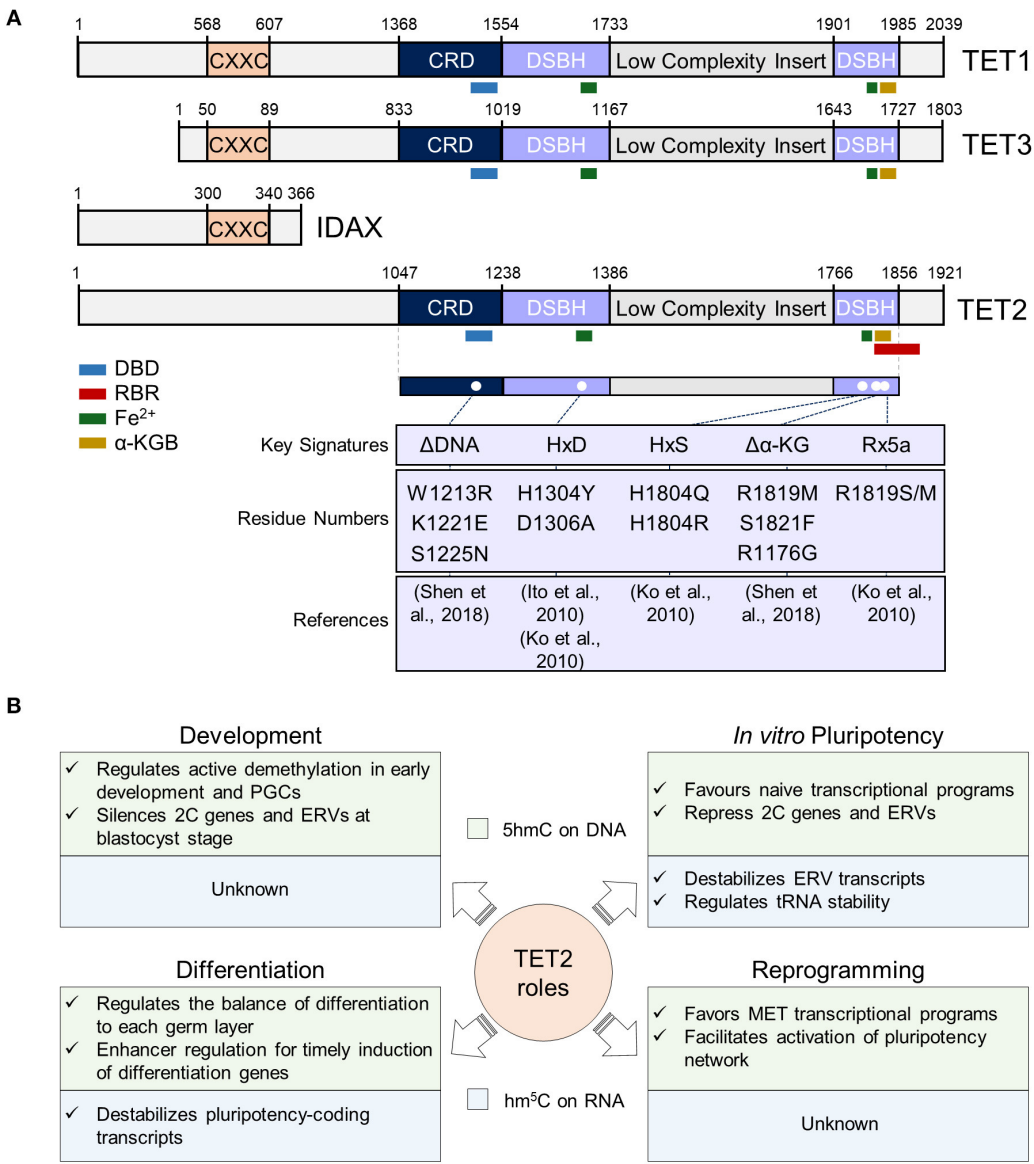
Overview of TET2 structure and functions in different cellular contexts. **(A)** TET2 structure in the context of TET proteins. In the top panel, sequences of mouse TET1 (NP_001240786.1), TET2 (ACY38292.1), TET3 (NP_001334242.1), and IDAX (NP_001004367.2) proteins are represented. The numbers represent the amino acid numbers. The two conserved domains cysteine-rich (CRD) and double-stranded beta-helix (DSBH) domains are indicated [based in Hu et al. (2013) and Iyer et al. (2009) alignments]. The conserved CXXC domain is also represented (coordinates were obtained from NCBI database). The TET2 RNA Binding Region (RBR) sequence described by He et al. (2016), was mapped first in the isoform 1 of TET2 (NP_001035490.2), and then in ACY38292.1 TET2 sequence. The interaction sites with DNA (DBD), RNA, Fe^2+^ and α-Ketoglutarate (α-KG) cofactors are also represented, and their sequences, also shown by Ito et al. (2010) and Hu et al. (2013), were obtained from the NCBI database. In the lower panel, described mouse TET2 mutations are shown. Since the indicated mutations have been mapped in the ACY38292.1 sequence, possible discrepancies may appear in the mutated positions according to the reference article. **(B)** Summary of TET2 functions in pluripotency and during development, differentiation, and somatic cell reprogramming. TET2 functions in these processes at the DNA and RNA level is indicated in the light green and blue squares, respectively. PGCs, primordial germ cells; 2C, 2-cell embryo; ERVs, endogenous retroviruses; MET, mesenchymal-to-epithelial transition.

TET2 protein contains an amino-terminal domain and a C-terminal catalytic domain which consists of a Cys-rich region and a double-stranded β helix (DSBH) with a large low-complexity insert (Figure 1A). Contrary to TET1 and TET3, during evolution TET2 lost its CXXC zinc finger domain, involved in binding of unmethylated CpG sequences, through a chromosomal inversion that resulted in the appearance of a new gene: IDAX (also known as CXXC4) (Iyer et al., 2009, 2011) (Figure 1A). Possibly due to the absence of a CXXC domain, TET2 is more associated to gene bodies and enhancers than to CpG-rich promoters (Hon et al., 2014; Huang et al., 2014). The DSBH domain contains key residues for the interaction of TET2 with its cofactors Fe(II) and 2-oxoglutarate (An et al., 2017) that are required for its catalytic function (Figure 1A). The interaction of TET2 with DNA has been investigated through the crystallization of the catalytic domain of human TET2 (TET2 CD) with 5mC-, 5hmC-, and 5fC-modified DNA (Hu et al., 2013, 2015). Importantly, TET2 catalytic cavity does not discriminate between 5mC and its oxidative derivatives, thus allowing iterative oxidation steps (Hu et al., 2013, 2015). Mutations introduced in TET2 DNA-interacting key residues or cofactor binding sites [i.e., Fe(II) and α-KG] have been shown to abolish its catalytic activity *in vitro* and *in vivo* (Ito et al., 2010; Ko et al., 2010; Hu et al., 2013; Shen et al., 2018) (Figure 1A). Although the minimal catalytically active fragment of TET2 is located in its C-terminal domain, the full-length protein shows a higher activity on DNA than an N-terminal truncation, suggesting important functions of the N-terminus of TET2 for its catalytic function (Hu et al., 2013; He et al., 2016). Indeed, not only the C-terminus, but also the N-terminal domain of TET2 was shown to be heavily post-translationally modified (Bauer et al., 2015; An et al., 2017), pointing to important regulatory functions of this region in TET2 regulation and function on DNA.

Besides its binding to DNA, more recently TET2 has also been shown to interact with RNA *in vitro* and *in vivo* (Fu et al., 2014; Guallar et al., 2018; Shen et al., 2018; He et al., 2020; Lan et al., 2020). Indeed, and in line with the observation that TET2 is less strongly associated to chromatin than TET1 (Vella et al., 2013), its recruitment to chromatin was recently shown to be facilitated by RNA and decreased upon global transcriptional inhibition (Guallar et al., 2018). While TET2 interaction with RNA was reported to be dependent on an RNA-binding region (RBR) (He et al., 2016) (Figure 1A), the absence of the RBR in a C-terminal truncation of TET2 shows reduced, but not fully abrogated TET2-RNA interaction (He et al., 2016). In line with this, TET2 interaction with RNA is greatly reduced in ESCs in absence of one of its most confident interactors: the RNA binding protein PSPC1 (Guallar et al., 2018). It is plausible then that TET2 possesses the intrinsic ability to bind RNA but requires intermediate RNA-binding interactors for stabilizing this interaction and allowing m^5^C oxidation. It remains to be determined whether the catalytic activity of TET2 on RNA is only dependent on its RBR or on other protein domains that have not been identified yet. Of note, TET2 presents a greater affinity *in vitro* for m^5^C than hm^5^C (Guallar et al., 2018), similar to what happens with DNA (Hu et al., 2015). Although TET2 DNA-binding domain (DBD) and RBR domain have been mapped to different regions of the protein (Figure 1A) both were found to fold in proximity in human TET2 (Hu et al., 2013). Thus, it will be important to address how TET2 affinity and preference for RNA or DNA is regulated in the context of chromatin, where both nascent RNAs and genomic DNA can be oxidized (Chen et al., 2013; Hon et al., 2014; Guallar et al., 2018; Lan et al., 2020). Importantly, overexpression of full-length TET2 in HEK293T cells showed no catalytic activity in m^5^C in RNA, in contrast to TET2 catalytic domain, suggesting negative regulatory functions of the N-terminal domain on TET2 function on RNA oxidation (Fu et al., 2014). On the other hand, Hu and colleagues showed that the GS-linker which replaced the low complexity insert of the TET2-DNA in the crystal structure, was located distant from the core catalytic structure, suggesting that the low complexity insert is positioned on the exterior surface of the catalytic domain (Hu et al., 2013). Low complexity domains have been previously found in RNA-binding proteins and are involved in the formation of liquid-liquid phase-separation (LLPS) droplets by allowing multivalent and weak interactions (Franzmann and Alberti, 2019; Roden and Gladfelter, 2020). Given TET2's ability to interact with RNA, it is tempting to speculate that its low complexity insert could introduce a new layer of regulation through the formation of LLPS condensates. Future work will address this intriguing possibility and will determine the precise contribution and regulation of the different protein domains of TET2 to its roles as an epigenetic and epitranscriptomic regulator.

## TET2 Function in Early Development: Do We Know Everything?

Epigenetic modifications, such as DNA methylation and hydroxylation, are critical for normal early embryonic development (Monk et al., 1987; Howlett and Reik, 1991; Kafri et al., 1992; Hajkova et al., 2002; Guibert et al., 2012). During early embryogenesis, although readily detectable at the zygote, TET2 expression peaks in the inner cell mass (ICM) of the preimplantation blastocyst (Tang et al., 2010; Gu et al., 2011; Wossidlo et al., 2011). After implantation, TET2 expression is downregulated and is later only detected in primordial germ cells (PGCs) and in several adult tissues (Ito et al., 2010; Hackett et al., 2013; Guallar et al., 2018). Different groups have generated *Tet2* mutant mice, all consistently reporting that mice lacking TET2 are viable and fertile and can give rise to litters with normal size compared to their wild type counterparts (Ko et al., 2011; Li et al., 2011; Quivoron et al., 2011) (Table 1). Several studies have pointed to potential compensatory effects of different TET members during early embryogenesis, based on the observation that single *Tet1* or *Tet2* knock-outs are viable while combined loss of both enzymes in embryos leads to mid-gestation abnormalities and perinatal lethality (Dawlaty et al., 2013; Dai et al., 2016), though a substantial fraction of them being viable and fertile (Dawlaty et al., 2013). Given that no upregulation of *Tet1* or *Tet3* was observed upon *Tet2* depletion in mice (Li et al., 2011; Quivoron et al., 2011), it remains to be determined how TET1/3 are relocated to cover for TET2 loss for epigenetic and/or epitranscriptomic regulation *in vivo*. Despite these observations, no detailed analysis has been performed on the effects of *Tet2* depletion at the earliest cell fate transitions *in vivo*. Indeed, although *Tet2* silencing with siRNAs in the zygote was compatible with normal development *in vitro*, it leads to an abnormal upregulation of 2-cell (2C) embryo-specific genes and endogenous retroviral elements up to the preimplantation blastocyst stage in mouse (Guallar et al., 2018) (Figure 1B). Aberrant control of endogenous retrovirus at critical stages during development can lead to mobilization of these genomic parasites with implications on genomic stability and regulation (Rebollo et al., 2012; Faulkner and Garcia-Perez, 2017). These data thus support that a detailed analysis of TET2 targets and function during early preimplantation development is needed to study previously unappreciated fine-tuning functions of this protein which could be relevant for later generations. Later on in development, TET2 participates, together with TET1, in the active DNA demethylation at PGCs, and their deletion leads to defects in 5mC erasure at imprinting sites, as well as LINE1 and IAP endogenous retroviruses *loci* (Hackett et al., 2013) (Figure 1B). Nevertheless, the specific contribution of TET2 to this process is waiting to be addressed.

**Table 1:** Overview of TET2 studies deciphering its role and functions in mouse, murine embryonic stem cells (mESCs), human embryonic stem cells (hESCs) and during somatic cell reprogramming.

**Model**	**Genotype**	**Phenotype/most relevant findings**	**References**
Mouse	*Tet2* KO	- Reduction in 5hmC levels in the genomic DNA of BM cells - 1/3 of *Tet2^−/−^ and 8% of Tet2^+/−^*died within 1 year of age - Development of myeloid malignancies	Li et al., 2011
	*Tet2* KO	- Amplification and competitive advantage of hematopoietic stem and progenitor cells - Pleiotropic alterations of the hematopoietic compartments including both lymphoid and myeloid lineages - Myeloid malignancies with differentiation abnormalities	Quivoron et al., 2011
	*Tet2* KO	- No evidence for embryonic lethality - Decreased genomic levels of 5hmC - Increase in the size of the HSPC pool in a cell-autonomous manner. - Competitive advantage of HSCs, leading to an enhanced hematopoiesis into both lymphoid and myeloid lineages.	Ko et al., 2011
	*Tet2* KD (Early embryo)	- No observable delay in embryonic development - Derepression of MERVL elements and MERVL-associated genes	Guallar et al., 2018
mESCs	*Tet2* KD	- ~40% of decrease in genomic 5hmC levels - No changes in ESC maintenance - Formation of large hemorrhagic and aggressive teratomas, with greater contribution from neuroectoderm.	Koh et al., 2011
	*Tet2* KD*/*KO	- ~50% of decrease in genomic 5hmC levels in *Tet2*-deficient mESCs - Loss of 5hmC in gene bodies and increased 5hmC levels at promoter/TSS regions in *Tet2*-deficient mESCs - No detectable 5hmC levels in *Tet2*^−/−^ mESCs	Huang et al., 2014
	*Tet2* KO	- >90% loss of global 5hmC levels - Enhancers hypermethylation - Delay in early differentiation markers expression	Hon et al., 2014
	*Tet2* KD	- Telomere shortening - Chromosomal instability	Yang et al., 2016
	*Tet2* KO	- Significant decrease in global 5hmC levels in both *naïve* ESCs and primed EpiLCs	Mulholland et al., 2020b
	*Tet2* KO	- Significant decrease in global 5hmC levels in *naïve* ESCs - Significant increase in DNA methylation at LINE1/L1 elements - Severe hypermethylation at promoters, gene bodies, and repetitive elements - Premature repression of *Dppa3*	Mulholland et al., 2020a
hESCs	*Tet2* KD	- Increased expression of neuroectoderm markers and decreased expression of mesoderm and endoderm makers during EBs differentiation - No detectable alterations in pluripotency	Langlois et al., 2014
Somatic cell reprogramming	*Tet2* KD *MEF+OSKM*	- Abolishment of iPSC generation - Decrease of H3K4me2 at pluripotency loci - TET2 binds to *Nanog* and *Esrrb loci* during SCR and catalyze 5hmC	Doege et al., 2012
	*Tet2* KD *mEGC+hB cell fusion*	- Reduction of ~ 50% in reprogramming capacity - Decrease in 5hmC levels at the somatic *Oct4* promoter	Piccolo et al., 2013
	*preiPSC* *+NANOG*	- TET2 overexpression enhances NANOG-mediated reprogramming - TET2 KD abolishes the reprogramming synergy of NANOG with a catalytically deficient mutant of TET1	Costa et al., 2013
	*Tet2* KO *MEF+OSKM*	- Reduction of ~70% in reprogramming capacity	Hu et al., 2014
	B cell^+^ CEBPα^+^ OSKM	- TET2 overexpression enhances SCR - CEBPα activates *Tet2* expression - TET2 binds and demethylates pluripotency loci (e.g. *Oct4*)	Di Stefano et al., 2014
	*Tet2* KO B cell+ CEBPα+ iOSKM	- TET2 is required for B cell activation by CEBPα and for reprogramming by OSKM - TET2 interactors (i.e. CEBPα, KLF4, TFCP2l1) contribute with TET2 to reprogramming	Sardina et al., 2018
	*2^*nd*^ MEF+ iOSKM*	- ZSCAN4F binds to TET2 and recruits it to 5hmC-modify genes involved in glycolysis and proteasome activity	Cheng et al., 2020

*Model of study, genotype, phenotype, and mayor findings are included along with the reference article. HSCs, hematopoietic stem cells; HSPCs, hematopoietic stem progenitor cells; KD, knockdown; KO, knockout; iPSCs, induced pluripotent stem cells. 2nd MEF, secondary system expressing inducible OSKM*.

Most importantly, loss of function of *Tet2* in mice results in abnormal DNA hydroxymethylation patterns in bone marrow cells and leads to the emergence of myeloid malignancies between 2 and 4 months of age (Li et al., 2011; Moran-Crusio et al., 2011; Quivoron et al., 2011; Mulholland et al., 2020a). In depth discussion of TET2 role in normal and malignant hematopoiesis can be found in several excellent reviews (An et al., 2017; Bowman and Levine, 2017; Lio et al., 2019). Moreover, TET2 deficient mice show a mortality of around one-third in homozygosity and an 8% in heterozygosity within the first year of life (Li et al., 2011), implying other yet uncharacterized key roles of TET2 during aging. Additionally, TET2 has been shown to play a role in adult stem cell maintenance and differentiation (Ko et al., 2010, 2011; Li et al., 2011, 2017; Moran-Crusio et al., 2011; Quivoron et al., 2011; Zhang et al., 2016; Gontier et al., 2018; Yang et al., 2018). Future work with single-cell 5hmC/hm^5^C mapping at the DNA and RNA level, together with the identification of TET2 DNA and RNA targets during development, tissue homeostasis and aging will shed light into unknown functions of TET2.

## An Expanded View of TET2 in Pluripotency *in vitro*

Pluripotent embryonic stem cells (ESCs) of mouse origin are derived from the ICM of the preimplantation blastocyst (Evans and Kaufman, 1981; Martin, 1981) and can be maintained indefinitely *in vitro* without affecting their properties of self-renewal and differentiation potential. When maintained in conventional culture conditions (i.e., serum/LIF), ESCs are a heterogeneous population of cells with different developmental potential, including cells with totipotency-like features and the duet of *naïve* and primed pluripotent subtypes (Weinberger et al., 2016; Li and Izpisua Belmonte, 2018). Thus, ESCs undoubtfully represent a useful *in vitro* model to study molecular events taking place during early development, and a great amount of the data accumulated during these years regarding how pluripotency is governed have been derived from investigating ESCs. TET2 expression is high in pluripotent ESCs and decreases during differentiation (Ficz et al., 2011; Koh et al., 2011; Hon et al., 2014). Indeed, *Tet2* gene in ESCs is bound and activated by the master pluripotency factor OCT4 (i.e., *Pou5f1*), with *Oct4* silencing causing a reduction of *Tet2* expression (Koh et al., 2011; Wu et al., 2013). Nevertheless, *Tet2* depletion does not affect ESC colony morphology or pluripotent marker gene expression and only moderately induces some differentiation markers such as *Pax6, Neurod1* or *Lefty1/2* which is compatible with pluripotency maintenance (Ito et al., 2010; Koh et al., 2011).

As mentioned above, ESCs cultured in presence of serum/LIF are heterogenous, and include a small population (<5%) of cells with an expanded potency (Macfarlan et al., 2012), known as 2C-like cells (2CLCs). These 2CLCs, in contrast to pluripotent stem cells, have the ability to contribute to the trophectoderm. 2CLCs present a similar transcriptome and proteome to that of cells of the 2C-embryo, including high levels of endogenous retroviruses, particularly MERVL elements (Macfarlan et al., 2012; Eckersley-Maslin et al., 2016). TET2 is required for proper silencing of 2C-genes and MERVL elements in ESCs, and its depletion leads to an increase of 2CLCs population *in vitro* (Guallar et al., 2018). Importantly, TET2 contributes to both transcriptional and epitranscriptomic regulation of MERVL elements by recruiting HDACs to chromatin and oxidizing m^5^C in MERVL transcripts, respectively (Figure 1B). Additionally, a very recent study by He et al. (2020) has shown that TET2 can also modify tRNAs to modulate tRNA fragmentation. Given that tRNA fragments have been previously involved in ERV control (Schorn et al., 2017), it is tempting to speculate that TET2-mediated hm^5^C deposition on tRNA contributes to ERV regulation in pluripotent cells. This exciting possibility remains to be addressed. On the other hand, we and others have shown that TET1 and TET2 not only display specific patterns of expression in defined pluripotent subtypes (Fidalgo et al., 2016; Pantier et al., 2019; Mulholland et al., 2020b), but most importantly, play opposite roles in regulating *naïve* and primed pluripotency (Fidalgo et al., 2016). While TET1 is important for primed pluripotency, TET2 is a key determinant of *naïve* one. In fact, *Tet2* overexpression in primed post-implantation epiblast stem cells (EpiSCs) is sufficient to restore *naïve* pluripotency (Fidalgo et al., 2016). Thus, exquisite control of TET proteins during early development has been revealed through the study of their function in ESCs, exposing a critical role for TET2 in limiting previous developmental stages in ESCs (i.e., 2CLCs) and defining the unique pluripotency features of *naïve* pluripotency. Future studies are needed to address how TET2 catalytic-dependent and independent activities orchestrate pluripotency heterogeneity on DNA and RNA regulatory layers.

## TET2 Role in Differentiation: The DNA and RNA Sides of the Story

In contrast to TET2 dispensability for ESC maintenance, it is at the exit of pluripotency that a negative effect of loss of *Tet2* has been documented in a variety of systems. On the one hand, *Tet2* depleted ESCs give rise to large hemorrhagic teratomas that grow more aggressively than controls and are accompanied by a greater neuroectoderm contribution, thus reflecting a skewed differentiation potential (Koh et al., 2011). On the other hand, absence of *Tet2* during differentiation of ESCs to neural progenitors (NPCs) leads to a delay in the induction of differentiation genes, molecularly explained by a gain in 5mC at enhancers in absence of TET2 activity (Hon et al., 2014) (Figure 1B). This lack of phenotype of *Tet2* absence in pluripotency maintenance but its requirement for proper differentiation has also been shown to be conserved in human ESCs (Langlois et al., 2014), supporting an evolutionary conserved requirement of TET2 for cell commitment.

Remarkably, although both *Tet1* and *Tet2* are highly expressed in ESCs, many studies show a higher contribution of TET2 to 5hmC levels of genomic DNA (Hon et al., 2014; Huang et al., 2014; Mulholland et al., 2020b). At the molecular level, TET1 and TET2 have been shown to target different genomic regions, and thus be responsible for the regulation of 5mC at distinct targets (Chen et al., 2013; Hon et al., 2014; Huang et al., 2014). In particular, TET2 contributes to enhancer hypomethylation by 5mC oxidation, which is important for timely induction of lineage genes upon differentiation (Hon et al., 2014) (Figure 1B). Moreover, TET2-mediated deposition of 5hmC in gene bodies in ESCs has been related to exon inclusion/exclusion regulation (Huang et al., 2014) (Table 1). These observations, together with the absence of *Tet1* upregulation upon *Tet2* depletion in ESCs, and *vice versa*, (Koh et al., 2011; Huang et al., 2014), point toward non-redundant functions of TET1/2 in pluripotency maintenance and exit (Huang et al., 2014; Mulholland et al., 2020b). This is in sharp contrast to the absence of strong phenotypes in embryonic development in single *Tet1* or *Tet2* knock-out animals, which only becomes obvious when both TET1/2 proteins are absent and a partially penetrant perinatal lethality appears (Dawlaty et al., 2013). Intrinsic differences of *in vitro* and *in vivo* studies may be behind these apparently contradictory observations. It may be as well possible that the effects of TET1 or TET2 individual loss-of-function may only be visible *in vivo* after a number of generations. Indeed, this could be expected according to several studies which reported defects in telomere maintenance in ESCs in absence of one or several of the TET members (Lu et al., 2014; Yang et al., 2016), which would have minor effects on F0-F1 offsprings, but could lead to the appearance of genomic instability and propensity to cancer development in later generations.

Besides its well-known presence on DNA as an epigenetic mark, hm^5^C has also been detected in RNA of ESCs (Fu et al., 2014; Guallar et al., 2018; Lan et al., 2020). Besides 2C-specific transcripts and MERVL RNAs (Guallar et al., 2018), around 800 mRNAs, including more than 100 pluripotency-related transcripts, are hm^5^C-modified in ESCs (Lan et al., 2020) (Figure 1B). Importantly, characterization of flag-tagged endogenous TET1 and TET2 RNA interactome revealed overlapping and distinct targets, suggesting common and specific roles of TET proteins on RNA regulation (Lan et al., 2020). Deletion of TET2 RNA-binding region (RBR) (He et al., 2016) resulted in a 47% decrease in TET2 interaction with ESC transcripts, supporting that additional TET2 domains and/or interactors, such as PSPC1, could be required for its stable interaction with RNA (Guallar et al., 2018; Lan et al., 2020). In line with the observation that transcriptional inhibition greatly reduces TET2 chromatin occupancy (Guallar et al., 2018), hm^5^C modified-RNAs were found to be enriched in nuclear nascent-chromatin associated RNAs, pointing to a co-transcriptional mechanism of RNA modification by TETs (Lan et al., 2020). In sharp contrast, Legrand et al. (2017) did not detect hm^5^C modification in mRNAs when analyzing total RNA in ESCs. A potential explanation could be that hm^5^C-modified RNAs are likely less abundant compared to their non-modified counterparts, given that this modification has been linked to RNA destabilization (Guallar et al., 2018; Shen et al., 2018; Lan et al., 2020), and thus more difficult to detect. Future studies will be needed to further clarify these apparent contradictions. Importantly, RNA destabilization of pluripotency-coding transcripts, through m^5^C oxidation to hm^5^C including some encoding for Polycomb proteins, is important for contributing to transcriptome flexibility necessary for differentiation (Lan et al., 2020). This is in agreement with previous observations in both pathological and physiological contexts which have reported a stabilizing role of m^5^C on mRNA (Chen et al., 2019; Yang et al., 2019). Future studies are required to interrogate hm^5^C patterns and dynamics during early development, coinciding with TET expression, and its potential role in fine-tuning cell fate transitions through mRNA modifications.

## TET2 Functions in the Establishment of Pluripotency

The process of somatic cell reprogramming (SCR) to regain pluripotency from somatic cells was first achieved by overexpressing four transcription factors (*Oct4, Sox2, Klf4*, and c*-Myc*) called OSKM or Yamanaka factors (Takahashi and Yamanaka, 2006). This complex process of cell fate transitions leads to the generation of induced pluripotent stem cells (iPSCs) and involves wide remodeling cell fate steps, including the shutting down of somatic transcriptional programs and reactivation of pluripotency genes (Apostolou and Stadtfeld, 2018), and a metabolic rewiring toward a more glycolytic and less oxidative cellular metabolism (Yoshida et al., 2009; Zhu et al., 2010; Folmes et al., 2011; Mathieu et al., 2014). *Tet2* expression is induced as early as day 2 of OSKM reprogramming in mouse embryonic fibroblasts (MEFs) (Doege et al., 2012). TET2 has been reported to play key roles in a myriad of reprogramming systems, which include reprogramming of adult B cells through cell fusion (Piccolo et al., 2013), pre-iPSC reprogramming by NANOG (Costa et al., 2013), B cell reprogramming through CEBP and OSKM overexpression (Di Stefano et al., 2014; Sardina et al., 2018) and the classical Yamanaka reprogramming (Doege et al., 2012; Hu et al., 2014; Cheng et al., 2020) (Table 1). All these studies reflect a critical role of TET2 as an epigenetic regulator for pluripotency reacquisition in somatic cells. At the molecular level, TET2 has been shown to be important for 5hmC deposition on DNA and transcriptional regulation of pluripotency genes, mesenchymal-to-epithelial facilitators (i.e., miR200 cluster), proteasomal subunits and glycolytic regulators (Doege et al., 2012; Piccolo et al., 2013; Di Stefano et al., 2014; Hu et al., 2014; Sardina et al., 2018; Cheng et al., 2020) (Figure 1B and Table 1). Importantly, TET2 is required both at the earliest phases of reprogramming, and during later stages of pluripotency reacquisition, further underlining the relevance of TET2 as a central reprogramming player (Doege et al., 2012; Costa et al., 2013; Di Stefano et al., 2014; Hu et al., 2014; Sardina et al., 2018; Cheng et al., 2020). Importantly, TET2 has been shown to mediate its roles in reprogramming in coordination with several transcription factors including NANOG, CEBPα, KLF4, TFCP2l1, and ZSCAN4F (Costa et al., 2013; Sardina et al., 2018; Cheng et al., 2020), though for some of them definitive proof of direct recruitment has not been provided. Although at this stage there is no doubt of TET2 relevance for iPSC generation as a transcriptional regulator through DNA hydroxymethylation, its contribution to pluripotency reacquisition through RNA modification is currently unexplored and will need further studies.

## Summary and Implications

TET2 function as an epigenetic modifier through 5mC to 5hmC, 5fC and 5caC oxidation is currently fully accepted. The relevance of TET2-mediated catalytic-dependent functions, has been implicated *in vivo* in the correct function of hematopoietic compartment (Lio and Rao, 2019) and *in vitro* in the appropriate establishment of the epigenetic landscape that endows a correct timing of differentiation program expression as well as facilitates pluripotency reacquisition through SCR (Doege et al., 2012; Piccolo et al., 2013; Di Stefano et al., 2014; Hon et al., 2014; Hu et al., 2014; Langlois et al., 2014; Sardina et al., 2018). In contrast, only a handful of studies have shown the implication of TET2 in these physiological and pathological contexts in terms of RNA m^5^C oxidation (Figure 1B). The determination of the TET2-RNA complex structure, combined with the known TET2-DNA complex (Hu et al., 2013, 2015) may provide additional mechanistic insights into differences and similarities between TET-mediated oxidation of m^5^C/5mC in RNA and DNA. Moreover, neither the enzyme(s) responsible for depositing m^5^C on RNA for TET2 oxidation, nor the hm^5^C readers in charge of destabilizing TET2-targeted RNAs are currently known. Furthermore, the observation that a fraction of its interactome is comprised by unmodified RNAs raises the possibility that TET2 could also have catalytic-independent functions on RNA, similar to what has been previously described on DNA (Chen et al., 2013; Zhang et al., 2015; Guallar et al., 2018; Ito et al., 2019). The development of technology that allows mapping of chromatin regulators using low input samples, coupled with the appearance of sequencing techniques which can faithfully distinguish 5mC/m^5^C from 5hmC/hm^5^C at a single base resolution on DNA and RNA, respectively, opens up exciting possibilities to explore TET2 function as an epigenetic and epitranscriptomic regulator during early development, in ESC subpopulations and in tissue heterogeneity in the near future.

## Author Contributions

All authors listed have made a substantial, direct and intellectual contribution to the work, and approved it for publication.

## Conflict of Interest

The authors declare that the research was conducted in the absence of any commercial or financial relationships that could be construed as a potential conflict of interest.
